# Bioanalytical Performance of a New Particle-Enhanced Method for Measuring Procalcitonin

**DOI:** 10.3390/diagnostics10070461

**Published:** 2020-07-07

**Authors:** Anne Marie Dupuy, Anne Sophie Bargnoux, Romaric Larcher, Antoine Merindol, Thomas Masetto, Stéphanie Badiou, Jean Paul Cristol

**Affiliations:** 1 Laboratoire de Biochimie et Hormonologie, CHU Montpellier, Université Montpellier 1, F-34295 CEDEX 5 Montpellier, France; am-dupuy@chu-montpellier.fr (A.M.D.); as-bargnoux@chu-montpellier.fr (A.S.B.); a-merindol@chu-montpellier.fr (A.M.); s-badiou@chu-montpellier.fr (S.B.); 2 Laboratoire de Biochimie et Hormonologie, PhyMedExp, Université de Montpellier, INSERM, CNRS, CHU de Montpellier, 34295 Montpellier, France; r-larcher@chu-montpellier.fr; 3 Département de Réanimation, CHU Montpellier, Université Montpellier 1, F-34295 CEDEX 5 Montpellier, France; 4 Institute of Molecular Medicine I, Medical Faculty, Heinrich Heine University Düsseldorf, 40225 Düsseldorf, Germany; Thomas.Masetto@hhu.de

**Keywords:** PCT, correlation, precision, clinical concordance

## Abstract

We report the analytical performances of two particle-enhanced (PETIA) methods for measuring procalcitonin (PCT), the Diazyme PCT and the new DiaSys PCT assay, and their concordance of values with BRAHMS PCT Kryptor©. The total imprecisions onto two control levels and one serum pool were for DiaSys 5.42%, 3.3% and 7.53% and for Diazyme 10.7%, 2.9% and 13.23%, respectively. The limit of blank, limit of detection and limit of quantification were under the 0.25 cut-off for the two methods. The linearity in the lower range was acceptable for both methods. No significant effect on PCT determination was observed for DiaSys’ assay upon addition of interfering substances. With the Diazyme assay, significant effects were seen with rheumatoid factor (RF), lipid and hemoglobin. Correlation studies on 136 sera showed a good correlation between PCT measurements using DiaSys assay against the Kryptor system, while only a poor correlation was observed between the Diazyme assay, especially for low values. The novel PETIA PCT assay from DiaSys shows analytical performances acceptable for clinical use and the concordance with Kryptor method was fine at all clinical cut-offs. In contrast, despite comparable analytical performances, the Diazyme PETIA method exhibited a poor concordance with the Kryptor method.

## 1. Introduction

In the last decade, procalcitonin (PCT) has emerged as a useful biomarker in diagnosis and management of sepsis, and has become an essential parameter to differentiate bacterial from viral infections in different cohorts of patients [1,2]. Recently, the importance of PCT determination has also been highlighted for the prognosis of COVID-19 [3,4]. The availability of rapid, easy to apply and precise assay systems to check systemic inflammation are absolutely essential to diagnose and manage patients with follow-up infection for SARS-CoV-2. Meta-analyses have proven dramatically increased mortality in emergency patient populations when PCT values were higher than 0.25 µg/L and 0.5 µg/L [5]. Different cut-offs have been suggested for determining the bacterial origin of the potential infection and, as a consequence, the antibiotic stewardship. For respiratory tract infections or for meningitis, a bacterial origin is likely if the PCT value is greater than 0.25 µg/L, while for the intensive care unit, a bacterial infection is likely if the PCT is over 0.5 µg/L and very likely if the PCT level is over 1 µg/L [5,6,7]. In addition, PCT could be a helpful biomarker to reduce patient exposure to antibiotics without any significant effect on mortality [1,8]. A recent meta-analysis also suggests that procalcitonin-guided therapy could reduce mortality in critically ills [9]. All the clinical cut-off values have been defined according to the first available method for measuring PCT, the BRAHMS PCT assay. However, numerous assays for PCT determination have been developed and are available on different platforms in core labs or as point of care systems at the bedside. Some companies, for example Abbott, Biomérieux, Diasorin, Fujirebio, Roche and Siemens, use anti-PCT antibodies from BRAHMS. The main difference between these assays is the detection system. Recently, several companies have developed their own tests including Radiometer [10], Boditech [11], Diazyme [12,13,14] and more recently DiaSys, using polyclonal antibodies. The latter two use particle-enhanced turbidimetry (PETIA) and are available on open platforms. However, in the absence of standardization of PCT assays, the analytical performances of different kits should be tested and their concordance with the clinical cut-offs should be evaluated. The possibility of setting up a reliable immunoturbidimetric test has the advantages of being applicable to all the clinical chemistry analytical instruments, lower the costs and shorten the time-to-result.

Here, we report the analytical performances of the two PETIA assays for measuring PCT from Diazyme and DiaSys applied on a Roche Cobas8000© analyzer, and we evaluate the concordance with the BRAHMS PCT Kryptor CompactPlus© method, currently considered as the reference method. 

## 2. Material and Methods 

### 2.1. PETIA (Particle-Enhanced) Methods for PCT Measurements 

PETIA (particle-enhanced) methods were conducted using the c502 Cobas 8000© from Roche (Meylan, France). All reagents, calibrators, and quality controls were distributed by DiaSys company (Holzheim, Germany). The two PETIA methods required a 6-point calibration with quite similar levels between 0.80 to 50 µg/L (0.84, 3.49, 10.93, 23.56, 53.94 for DiaSys and 0.85, 2.85, 14, 23.79, 52.37 for Diazyme). PETIA methods have 2 levels of internal quality controls; level 1 is 0.93 and 1.5 and level 2 is 10.9 and 16 for DiaSys and Diazyme, respectively.

### 2.2. BRAHMS PCT Kryptor CompactPlus© Method for PCT Measurements 

PCT levels were determined using the BRAHMS© PCT assay, which is routinely used at the central laboratory. The BRAHMS© PCT assay is an approach based on time-resolved amplified cryptate emission (TRACE) that is conducted using the Kryptor Compact Plus© instrument (B.R.A.H.M.S. AG, Hennigsdorf, Germany), with an anti-calcitonin polyclonal antibody conjugated with europium cryptate and an anti-katacalcin monoclonal antibody conjugated with XL665. PCT measurements were obtained within 19 min on the Kryptor^®^ system. The limit of blank (LoB), limit of detection (LoD) and limit of quantification (LoQ) reported by the manufacturer were 0.01 µg/L, 0.02 µg/L and 0.06 µg/L, respectively. 

### 2.3. Analytical Performances of the PETIA PCT Immunoassays 

Imprecision studies were conducted in accordance to the CLSI EP15 protocol (five measurements per day on two levels for 5 consecutive days) [15] with quality control materials and with serum pools. Calibrators and controls from DiaSys are ready to use while those from Diazyme are lyophilized and need to be re-suspended before each daily application or aliquoted and frozen at −20 °C. The LoD was determined according to the current CLSI standards [16]. The LoQ was calculated according to the US FDA protocol consisting of 5 times the limit of blank (LoB) [17]. A serum pool with a PCT concentration of 5.64 µg/L was used to test linearity in the lower range. The pool was diluted with diluent provided by manufacturer to the following final concentrations: 2.82, 1.41; 0.56, 0.28, 0.14, and 0.03 µg/L. Each dilution was measured in duplicate.

### 2.4. Interference Studies on PETIA Methods for PCT Quantification

Based on serum with PCT concentration of 0.5–0.7 µg/L, the influence of rheumatoid factor, triglyceride content, conjugated and unconjugated bilirubin as well as hemolysis on PCT quantification were tested, following CLSI protocol EP 07-A2 [18]. High concentrated materials RF (rheumatoid factor; positive plasma, Biospacific, 5100 IU/mL), triglyceride extract (Technopath, 8000 mg/dl), unconjugated bilirubin (Sigma-Aldrich, St Louis, MO, USA) and conjugated bilirubin (Frontier Scientific, Logan, UT, USA) were spiked into human serum to obtain concentrated stock solutions. These stock solutions were individually mixed with non-spiked serum to gain different concentrations of interferent materials. To analyze hemolysis, washed human erythrocytes were lysed through a detergent. The hemoglobin concentration of the lysate was photometrically quantified using the hemoglobin (Hb) reagent (Bioanalytic GmbH, Umkirch, Germany). The lysate was then spiked into human serum to obtain the desired hemoglobin concentration.

The interference measurements were performed in triplicate on a Cobas c502 in parallel for DiaSys and Diazyme PCT reagents. The bias was determined as the difference between average of baseline values determined in pools free of interference (T0) and average of values obtained from each overload of pools (Tx). The percentage deviation from T0 was calculated using Equation $\left[\frac{Tx-T0}{T0}\right]\times 100$. The variation corresponding to the 10% changes (±10%Δ) was considered as significant. 

### 2.5. Comparison Studies

First, the comparison study between the PETIA methods and BRAHMS PCT Kryptor Compact Plus© assays was conducted using samples from 136 consecutive patients admitted to the Emergency and the Intensive Care Unit (ICU) departments of Lapeyronie university hospital (Montpellier, France) with values within the analytical range of 0.02–50 µg/L. After centrifugation, serum samples were analyzed once using a Kryptor CompactPlus© instrument and re-analyzed on Cobas8000© analyzer with the two methods, within 2 hours. No additional sampling was requested, and only residual samples were used for method comparisons of this study. The study was approved by a local ethical committee (DC-2009-1052). In the presence of discordant results between the two methods, the samples were re-analyzed on the two instruments and the clinical record of patients were examined.

Secondly, to compare the PETIA assays, the external quality assessment (EQA) specimens stored at −80 °C from 2017 and 2018 ProBioQual EQA program (ProBioQual, Lyon, France) were measured too. 

### 2.6. Statistical Analysis 

Passing–Bablok regression analysis was used to compare the results of the PETIA assays with the BRAHMS PCT Kryptor© assay. The scatter of differences was visualized by means of Bland–Altman plots [19]. The CUSUM test and the recommendations of Emerson et al. [20] were used to detect deviation from expected values in linearity studies. Statistical analyses were performed using XLSTAT^®^ software, version 2016.06.35661 (Addinsoft, Paris, France). 

The concordance between the methods for classification of patients according to clinical algorithms was assessed using Cohen’s κ-test on the population divided into four categories: PCT values < 0.25, 0.25–0.5, 0.5–1.99, ≥ 2.0 µg/L [21]. For all comparisons, *p*-value < 0.05 was considered statistically significant. 

## 3. Results

### 3.1. Analytical Performances

Analytical performances (imprecision, LoD, LoB, LoQ and linearity study) of the PETIA assays are presented in Table 1. While the CV (coefficient of variation) at level 2 was comparable for the two PETIA methods, the CV at level 1 from PCT Diazyme assay (10.07%) was twice higher than that of PCT DiaSys assay (5.42%). The same difference was found with the serum pool (7.53 vs. 13.23% for DiaSys and Diazyme, respectively). LoB, LoD and LoQ were always largely lower than 0.25 µg/L, regardless of the assay. 

The Passing–Bablok regression of linearity showed a gradual decrease in recovery from 100.0% on the highest sample measured with DiaSys assay (assigned value 7.08 µg/L) to 50.8% on the low sample (assigned value 0.18 µg/L). With Diazyme assay, a positive and increasing bias was observed starting from the sample with assigned value of 0.35 (recovery 148.7%) down to the last dilution. This positive bias that may derive from some interference could affect the specificity of the test.

The DiaSys assay displayed a more acceptable linearity than Diazyme’s over the most clinically relevant range for PCT (0.5 µg/L). The CUSUM test for both assays did not show significant deviation from linearity. However, the difference of recovery (%) was higher than 20% for both assays.

There were no significant effects on PCT values measured by DiaSys assay with the addition of RF, triglycerides, bilirubin (unconjugated and conjugated) and Hb. The variation in each spiked serum sample compared to the baseline sample remained much lower than 10%. No interference was observed with unconjugated and conjugated bilirubin with the Diazyme assay, as described in the technical data as well. However, the results of RF, lipid and Hb interference on the serum pool at low PCT level showed that the addition of low, mild and high concentration of these interferent materials do interfere with the results leading to a positive bias, not described by the manufacturer so far [22]. The corresponding figures are given in Supplementary Materials.

### 3.2. Correlations between PETIA and BRAHMS PCT Assays

**Figure 1:**
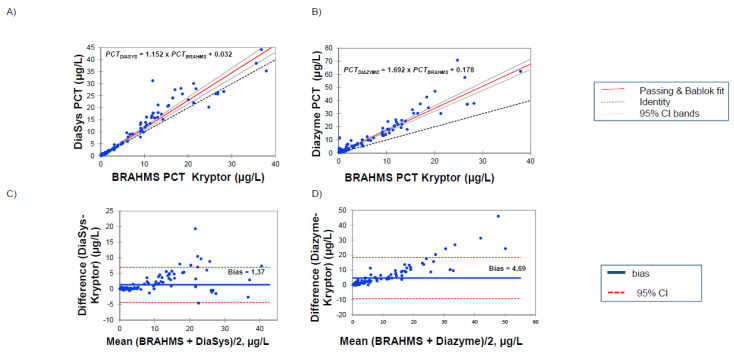
Passing-Bablok regression analysis on serum samples in the range 0.02–50 μg/L of BRAHMS PCT Kryptor against (**A**) DiaSys PCT and (**B**) Diazyme PCT assay. Bland-Altman analysis of BRAHMS PCT vs (**C**) Diasys PCT and (**D**) Diazyme PCT assay.

**Figure 2:**
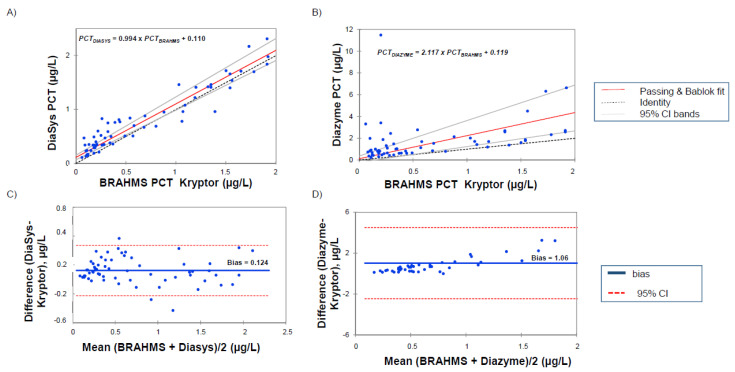
Passing-Bablok regression analysis on serum samples in the range 0.02–2 μg/L of BRAHMS PCT Kryptor against (**A**) DiaSys PCT and (**B**) Diazyme PCT assay. Bland-Altman analysis of BRAHMS PCT vs (**C**) DiaSys PCT and (**D**) Diazyme PCT assay.

The regression analyses between the PCT measurements of 136 samples on the Kryptor Compact Plus© and two PETIA methods are reported in Figure 1 and Figure 2. 

A clear correlation is obtained between DiaSys and Kryptor for the all range of measures with only a slight overestimation for the high levels (PCT*_DIASYS_* = 1.152 × PCT*_BRAHMS_* + 0.032, *p* < 0.002). The analysis of a low value, lower than 2 µg/L, confirmed the good correlation between the clinical cut-offs (PCT*_DIASYS_* = 0.994 × PCT*_BRAHMS_* + 0.110). These results were supported by Bland–Altman representation (bias at 1.37 µg/L in the entire measurement range and 0.124 µg/L for values lower than 2 µg/L). By contrast, results with the Diazyme assay exhibited a poor correlation with a clear overestimation in the entire measurement range (PCT*_DIAZYME_* = 1.692 × PCT*_BRAHMS_* + 0.178; *p* = 0.282). These results are particularly evident for PCT levels lower than 2 µg/L (PCT*_DIAZYME_* = 2.117 × PCT*_BRAHMS_* + 0.119; *p* = 0.952). Here, again, these results are confirmed by Bland–Altman representation (bias at 4.69 µg/L in the entire measurement range and 1.06 for values lower than 2 µg/L).

### 3.3. Concordance at the Cut-Off Levels

**Figure 3:**
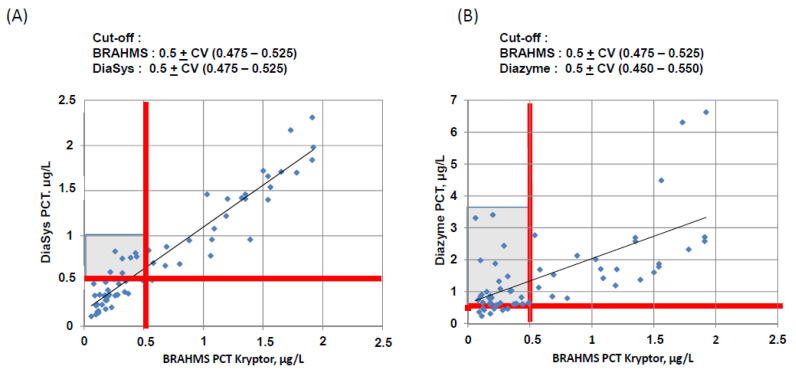
Correlation of BRAHMS PCT assay vs (**A**) DiaSys PCT assay and (**B**) Diazyme PCT assay in the range from 0.02 to 2 μg/L: particularly noteworthy is the over all discrepancy expressed for each method in terms of CV. The grey zone reflects discordant determinations of the two assays. The white zone reflects samples which are similarly analyzed by the two assays. For BRAHMS PCT, the CV was calculated from ThermoFisher internal quality control (IQC), used in our lab over a 12-month period. For DiaSys and Diazyme PCT, the CV was calculated in this study (see Table 1).

The overall discordance between the clinical cut-offs (0.25, 0.25–0.50, 0.50–2, > 2 µg/L) was found to be 19% with DiaSys assay and 38% for Diazyme assay. The Cohen’s Kappa were 0.695 [IC_95%,_ 0.605–0.786] and 0.401 [IC_95%,_ 0.306 to 0.496] for DiaSys and Diazyme, respectively. The detailed values in each category are reported in the Appendix A (Table A1). Then, we analyzed the discrepant results at the clinical cut-off of 0.5 µg/L (Figure 3) taking into account the CV observed for the respective PETIA system and claimed by the manufacturer for PCT Kryptor Compact Plus©. With DiaSys assay, only 9 discordants, with an overestimation, have been observed and the clinical chart revealed for three patients a potential infection (patient with suspected meningitis, one patient receiving antibiotics in ICU and sepsis case) (Table 2). A total of 21 discordants, with an overestimation, were observed with Diazyme assay. All the discordant results obtained with DiaSys assay were also found with Diazyme assay. However, among these 21 overestimations only 6 were suspected of bacterial infection (Table 2). Generally, there were 6 false negative results with BRAHMS assay, 2 false negative results with DiaSys assay and 15 false positive results with Diazyme assay vs. 6 with DiaSys assay. No false negative results were observed with Diazyme assay.

### 3.4. Analysis of the EQA Samples

**Figure A1:**
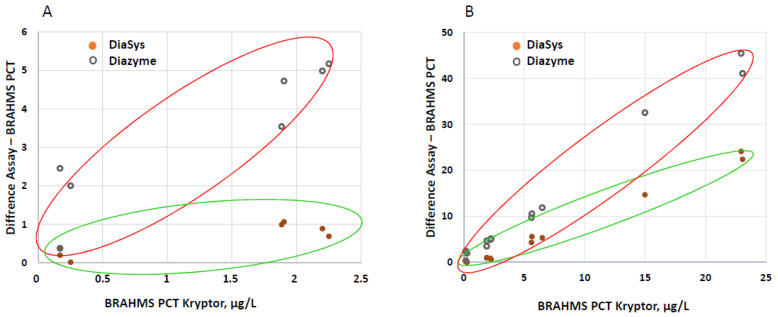
Schematic representation of results obtained with ProBioQual samples with BRAHMS PCT and DiaSys and Diazyme PCT (**A**) for low concentrations of EQA samples; (**B**) for high concentrations of EQA samples. X-axis indicates the BRAHMS PCT values obtained on EQA samples; Y-axis indicates the difference between values of DiaSys or Diazyme PCT and BRAHMS PCT values.

Using the EQA samples, PCT values with the two PETIA methods were always higher than those reported by Kryptor Compact Plus© instrument. In absence of a reference method we plotted the values observed with BRAHMS PCT Kryptor on the horizontal axis. The difference between PETIA assays and BRAHMS PCT assay for each sample is plotted on the vertical axis. A total of 13 different external controls are analyzed. Clearly, Diazyme assay demonstrates a huge overestimation in comparison to Kryptor, especially for EQA levels lower than 2 µg/L (see Figure A1 in Appendix A). 

## 4. Discussion

This study demonstrated that the analytical performances of the PETIA method from DiaSys reagents applied on c502 Cobas 8000© from Roche are acceptable for clinical use and that the DiaSys PCT significantly improves the precision at low PCT levels in comparison to Diazyme assay. However, it must be noted that both PETIA tests display an improvable linearity in the low range. It is noteworthy that the Diazyme assay exhibits a strong positive bias at these low concentrations, which does affect the diagnostic significance of the test. In addition, comparison studies highlighted a good agreement between DiaSys assay and BRAHMS PCT Kryptor while a clear overestimation is observed for Diazyme assay. Finally, our study on the EQA samples stressed the need for standardization of PCT assays.

### 4.1. Performance Assessment

The observed level of imprecision is in line with previous publications on PETIA methods. Indeed, the CVs at level 2 (11.8 µg/L for DiaSys and 15.2 µg/L for Diazyme) were around 3% as previously described for Diazyme assays on Beckman system [12] or on Cobas c702© [13]. However, discordant results are reported for low levels of controls. In agreement with our results, Dipalo et al. [12], reported a high CV (7.7%) for low level of PCT control (1.31 µg/L) while Yuan [23] and Ceriotti [13] reported a CV lower than 6%. Few data are available on serum pools with low levels. Here, again, Diazyme assay shows an intra-laboratory CV of 10.63% for a pool at 0.61 µg/L close to our results. To the best of our knowledge, this is the first report of DiaSys PCT assay. Precision of DiaSys for low level of controls or for low level of serum pools was better than those obtained with Diazyme assay. Despite different technical principles between the latex-enhanced immunoturbidimetric assay developed by DiaSys and the sandwich immunoassay using time-resolved amplified cryptate emission technology used by Kryptor, the CVs are closed and remained largely lower than 10% at every level of control or serum pool. 

The observed LoB, LoD and the functional sensitivity, defined as the lowest PCT concentration that could be analyzed with a coefficient of variation (CV) ≤ 20%, are in agreement with previous results obtained with PETIA methods [12,13,23]. Interestingly, all the LoQ are lower than the clinical cut-off of 0.25 and are closed to the reported LoQ for Kryptor PCT BRAHMS method. The absence of interference is of great interest due to the high requests for PCT by the emergency, ICU and pediatrics departments for which the samples are frequently hemolyzed, turbidimetric, icteric and with the presence of RF. There are only a few studies on interferences for PCT so far. Generally, no significant interference was reported with RF, lipid, bilirubin and Hb [24,25,26]. However, based on our observation about the endogenous interferences for the assay of Diazyme, its use in routine should be done with caution. 

### 4.2. Comparison with BRAHMS PCT Kryptor and Clinical Concordance

No reference methods for PCT quantitation are yet available, and comparison studies could be difficult to interpret. Since all the clinical cut-offs were defined according to the first fully automated PCT assay, the BRAHMS PCT Kryptor, we decided to analyze the correlation and clinical performances against this method. In the published reports to date with PETIA methods, only Diazyme assays were analyzed. Discordant results have been published according to the correlation studies. Some studies performed on the entire measurement range reported fine correlation with slopes ranging from 0.85 [14] to 1.1 [12] and a modest bias around 0.5 to 1 µg/L. More recently, Lippi et al. [27] reported an acceptable correlation between Diazyme and BRAHMS PCT but with a large bias resulting in an overestimation reaching 38%. Our results are in agreement with this observation, the mean bias observed with Diazyme assay in the entire measurement range (up to 50 µg/L) reaching 4.69 µg/L. By contrast, DiaSys PETIA resulted only in a modest overestimation, with the mean bias appearing limited to 1.37 µg/L. In addition and in agreement with the report of Ceriotti [13], only a poor correlation was observed for low values with Diazyme assay (Figure 1 and Figure 2) while a good correlation (PCT*_DIASYS_* = 0.994 × PCT*_BRAHMS_* + 0.110) with a small bias (0.124 µg/L) was observed with DiaSys assay. 

The clinical concordance at each clinical cut-off was further analyzed using Kappa coefficients. The global kappa coefficients were close to 0.7 for DiaSys and, consequently, the strength of agreement is considered to be very good. On the other hand, the global kappa coefficients for Diazyme is only 0.4 which could be considered weak. This weak concordance between Diazyme and BRAHMS assays could be due to the lack of standardization and differences in the calibration process. However, some discordant results leading to a 5-fold increase between BRAHMS/DiaSys results and Diazyme assay could be also due to a potential analytical interference. The analytical performances obtained with PCT Diazyme in our study are unexpected relative to previous data already published [12,23] or to the manufacturer’s claims. Several reasons may explain the decrease in bioanalytical performance of Diazyme assay. One of them could be that the analytical performance was performed on devices with not identical technical characteristics (c702 or c502 modules from Cobas Roche, AU5800 from Beckman, Vidas from Biomérieux). As suggested by Ceriotti et al. [13], it seems that a significant part of the differences at PCT concentrations <0.5 μg/L is explained by the high imprecision of Diazyme´s test, combined with the relevant calibration bias (−33.7%). This notion could also explain the important loss of linearity. In addition, a lot-to-lot variation of Diazyme reagent and/or calibrator could be a reason of the poor results of Diazyme, even though we did not prove this in our work. The differences in the PETIA DiaSys assay and BRAHMS PCT Kryptor method were minimal and the use of the DiaSys PCT method would be suitable for bacterial infection diagnosis and antibiotic stewardship. In fact, considering the clinical cut-off of 0.5, only 9 discordants with an overestimation were identified and 3 had a potential risk of infection. Thus, no under-treatment could result from the DiaSys assay in clinical use. 

### 4.3. Lack of Standardization

Using EQA samples shows a lack of comparability between results from PETIA, PCT assays and BRAHMS Kryptor assays. In fact, PCT values with PETIA methods were always higher than those reported by the group of Kryptor Compact Plus© instrument, clearly showing that standardization of calibration would be desirable. The same results have been previously observed with other assays [14] using BRAHMS antibodies such as Abbott, Diasorin, Siemens and Roche, or with other antibodies such as Radiometer. This result strongly suggests once again that the overestimation of Diazyme is much greater than with DiaSys assays. These differences in PCT EQA could be due not only to antibodies but also to matrix effects of EQA samples, differences in measurement methods or interferences. However, even using the same method, the results of EQA determined by Diazyme and DiaSys were largely different.

## 5. Conclusions

In summary, the novel PETIA PCT assay from DiaSys shows analytical performances acceptable for clinical use. In addition, a clear correlation was observed with the Kryptor BRAHMS assay, and the concordance with the Kryptor method was fine at all clinical cut-offs. In contrast, despite comparable analytical performances, the Diazyme PETIA method exhibited only a poor concordance with the Kryptor method and a clear overestimation. Moreover, the discordance between the PETIA systems observed with the EQA system clearly suggested that standardization of PCT assays and commutability of EQA materials are necessary steps to understand the lack of comparability between results obtained by different PCT assays.

## Figures and Tables

**Table 1:** Analytical performances of the PETIA (particle-enhanced turbidimetry) PCT (procalcitonin) methods on the c502 Cobas 8000© analyzer.

	DiaSys Assay		Diazyme Assay	
Total CV imprecision results	Mean, µg/L	CV, %	Mean, µg/L	CV, %
Control level 1	0.99	5.42	1.50	10.70
Control level 2	11.80	3.30	15.20	2.90
Serum pool	0.61	7.53	0.77	13.23
LoB	0.019		0.030	
LoD	0.053		0.140	
LoQ	0.095		0.150	
**Linearity**				
Theoretical values from DiaSys measurement, µg/L	Mean of observed values with DiaSys assay, µg/L (% of mean recovery)	Theoretical values from Diazyme measurement, µg/L	Mean of observed values with Diazyme assay, µg/L (% of mean recovery)	
7.08	7.08 (100.0)	6.99	6.99 (100.0)	
3.54	2.58 (72.8)	3.50	2.70 (77.2)	
1.77	1.17 (66.1)	1.75	1.25 (71.5)	
0.71	0.51 (72.0)	0.70	0.67 (95.8)	
0.35	0.19 (53.6)	0.35	0.52 (148.7)	
0.18	0.09 (50.8)	0.17	0.46 (263.2)	
0.04	0.03 (67.8)	0.04	0.41 (938.4)	

**Table 2:** Clinical data for the discrepant results and determination of false negative or positive results according to the assay.

Discordants	BRAHMS PCT Values, µg/L	PCT DiaSys Values, µg/L	PCT Diazyme Values, µg/L	Final Diagnosis
1	0.08	0.47	**0.73**	Infectious endocarditis ^a,d^
2	0.10	0.13	1.98	Pharmaco-resistant epilepsies ^c^
3	0.11	0.24	0.91	Cirrhosis ^c^
4	0.12	0.15	0.66	ARDS ^c^
5	0.15	0.24	1.00	Polytrauma ^c^
6	0.17	0.34	**0.8**	Bronchial surinfection ^a,d^
7	0.18	0.34	0.60	Repeated fall ^c^
8	0.18	0.49	0.87	ICU without Antibiotherapy ^c^
9	0.19	0.29	0.81	Leg fracture ^c^
10	0.22	**0.6**	**1.88**	Suspected meningitis ^a^
11	0.25	0.52	1.32	ARDS ^b,c^
12	0.26	0.34	0.64	Cirrhosis decompensation ^c^
13	0.26	0.83	1.09	Heart stroke ^b,c^
14	0.29	0.47	**2.44**	Complicated pneumonia with ARDS ^a,d^
15	0.32	0.75	1.48	ICU without Antibiotherapy ^b,c^
16	0.35	**0.50**	**1.03**	Sepsis from parietal origin ^a^
17	0.37	0.36	0.61	Complication following cardiac surgery ^c^
18	0.39	**0.76**	**0.63**	ICU with Antibiotherapy ^a^
19	0.43	0.81	0.82	Lung Adenocarcinoma ^b,c^
20	0.44	0.77	0.61	ICU without Antibiotherapy ^b,c^
21	0.49	0.51	0.65	Decompensated respiratory acidosis ^b,c^
				

^a^: false negative for the BRAHMS PCT assay. ^b^: false positive for the PCT DiaSys assay. ^c^: false positive for PCT Diazyme assay. ^d^: false negative for PCT DiaSys assay. ARDS, acute respiratory distress syndrome; ICU, intensive care unit, the bold and gray highlight represent the results overestimated of PCT DiaSys and Diazyme assays compared to BRAHMS PCT assay, which were suspected of bacterial infection.

## Supplementary Materials

The following are available online at https://www.mdpi.com/2075-4418/10/7/461/s1.

## Appendix A

Repartition of the PCT results in our laboratory: (i) BRAHMS PCT assay vs. PCT DiaSys assay and (ii) BRAHMS PCT assay vs. PCT Diazyme assay on the 136 serum samples.

N = number of subjects in each category.

**Table A1:** Repartition of the PCT results in our laboratory (i) BRAHMS PCT assay vs PCT DiaSys assay and (ii) BRAHMS PCT assay vs PCT Diazyme assay on the 136 serum samples.

(i)	**PCT DiaSys Assay**	**BRAHMS PCT Assay**				
		**PCT < 0.25**	**0.25 ≤ PCT < 0.5**	**0.5 ≤ PCT < 2**	**PCT ≥ 2**	**Kappa Test**
	PCT < 0.25	10	2	0	0	0.695 (95%CI, 0.605 to 0.786)
	0.25 ≤ PCT < 0.5	11	2	0	0	
	0.5 ≤ PCT < 2	2	9	25	0	
	PCT ≥ 2	0	0	2	73	
(ii)	**PCT Diazyme Assay**	**BRAHMS PCT Assay**				
		**PCT < 0.25**	**0.25 ≤ PCT < 0.5**	**0.5 ≤ PCT < 2**	**PCT ≥ 2**	**Kappa Test**
	PCT < 0.25	3	0	0	0	0.401 (95%CI, 0.306 to 0.496)
	0.25 ≤ PCT < 0.5	7	2	0	0	
	0.5 ≤ PCT < 2	15	12	14	0	
	PCT ≥ 2	4	2	12	65	
