# Supplementary material for: Bioanalytical Performance of a New Particle-Enhanced Method for Measuring Procalcitonin
Source: Diagnostics (Basel). 2020 Jul 7;10(7):461. doi: 10.3390/diagnostics10070461 (PMC7400370; doi:10.3390/diagnostics10070461)
Supplement: Supplementary file 1 [file diagnostics-10-00461-s001.pdf]

Supplementary Materials:

Figure S1. Interferences studies: Influence on PCT values measured by DiaSys or Diazyme methods of rheumatoid factor (A and B), triglyceride content (C and D), conjugated (E and F) and unconjugated bilirubin (G and H) as well as hemolysis (I and J).

A

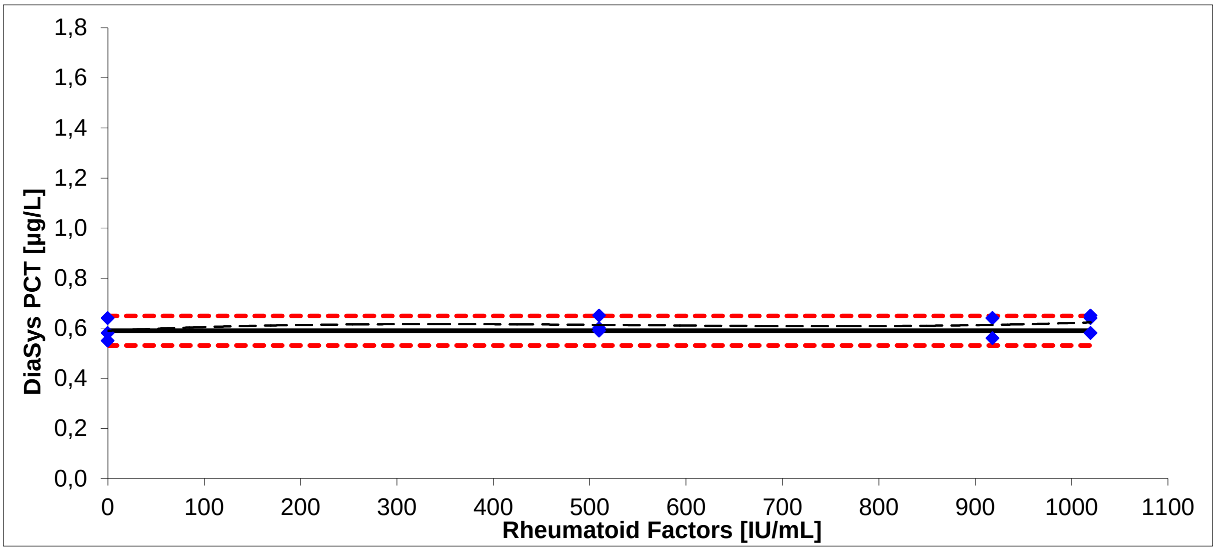

B

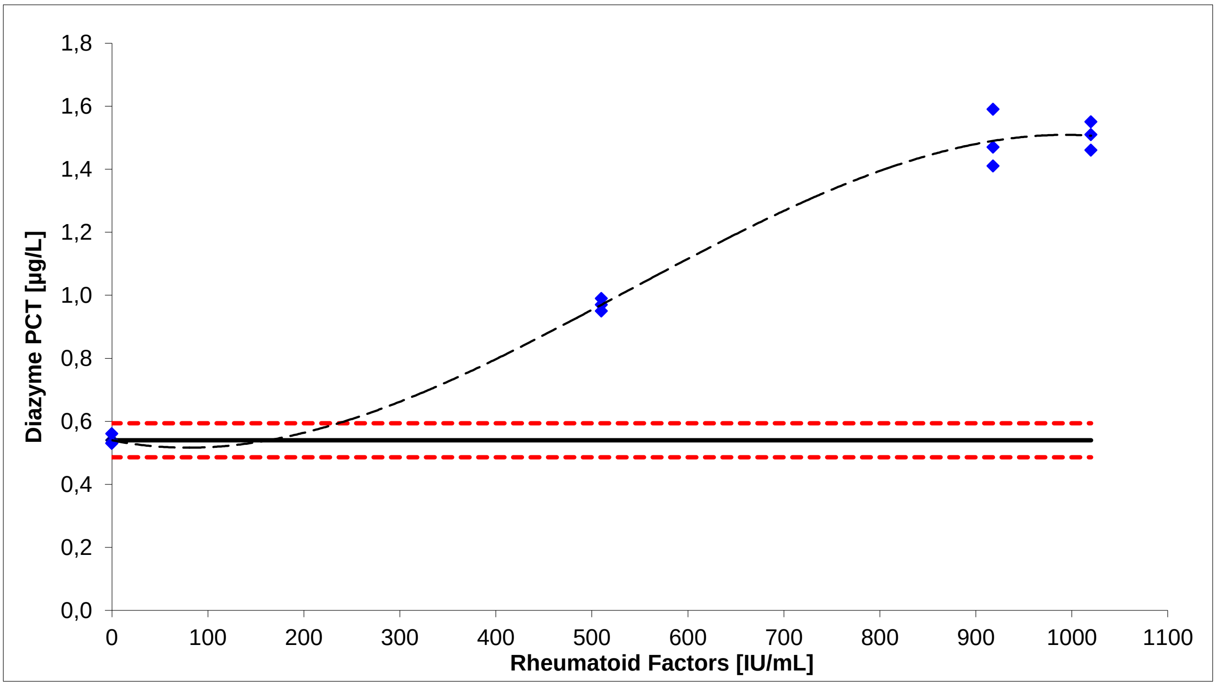

C

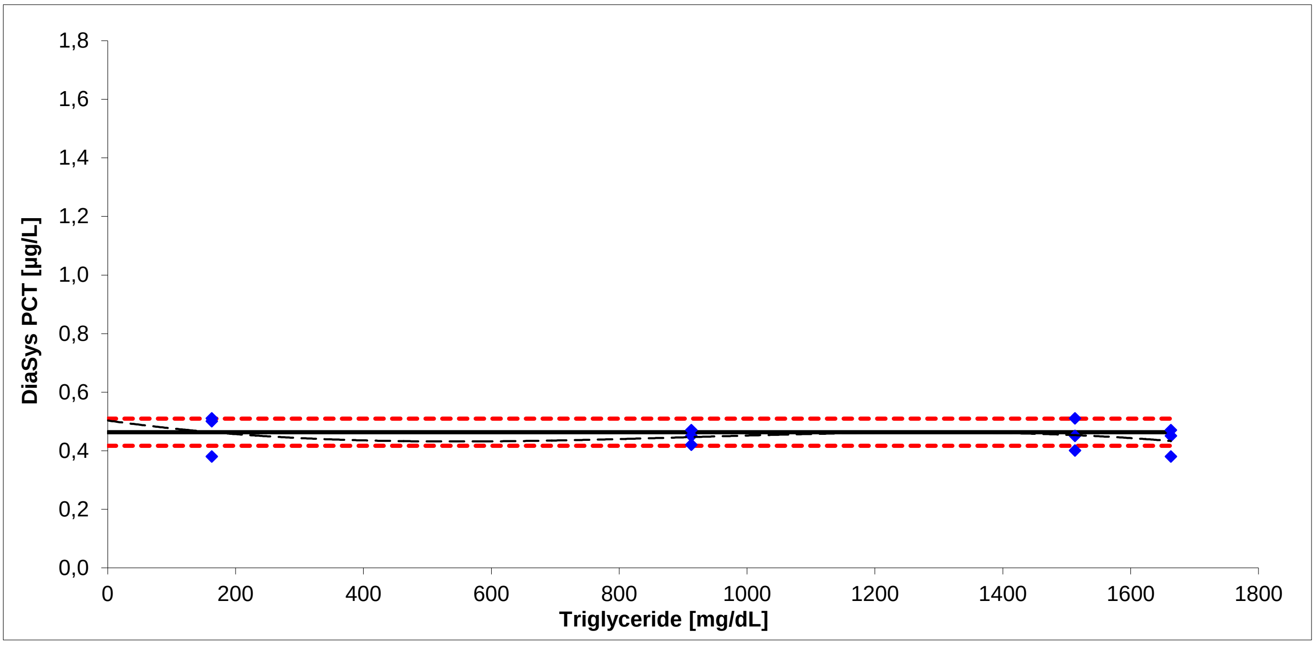
1,8
1,6
1,4
1,2
1,0
0,8
0,6
0,4
0,2
0,0
DiaSys PCT [µg/L]
0
200
400
600
800
1000
1200
1400
1600
1800
Triglyceride [mg/dL]

D

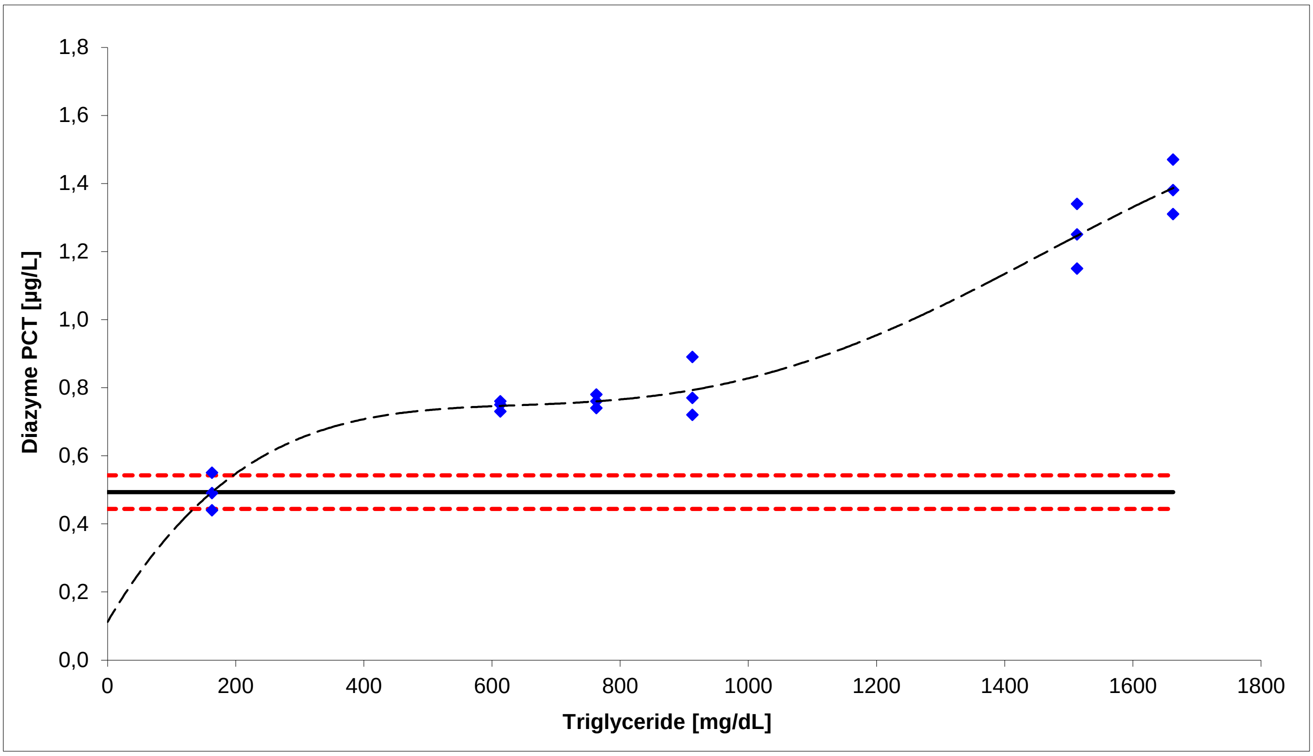
1,8
1,6
1,4
1,2
1,0
0,8
0,6
0,4
0,2
0,0
Diazyme PCT [µg/L]
0
200
400
600
800
1000
1200
1400
1600
1800
Triglyceride [mg/dL]

E

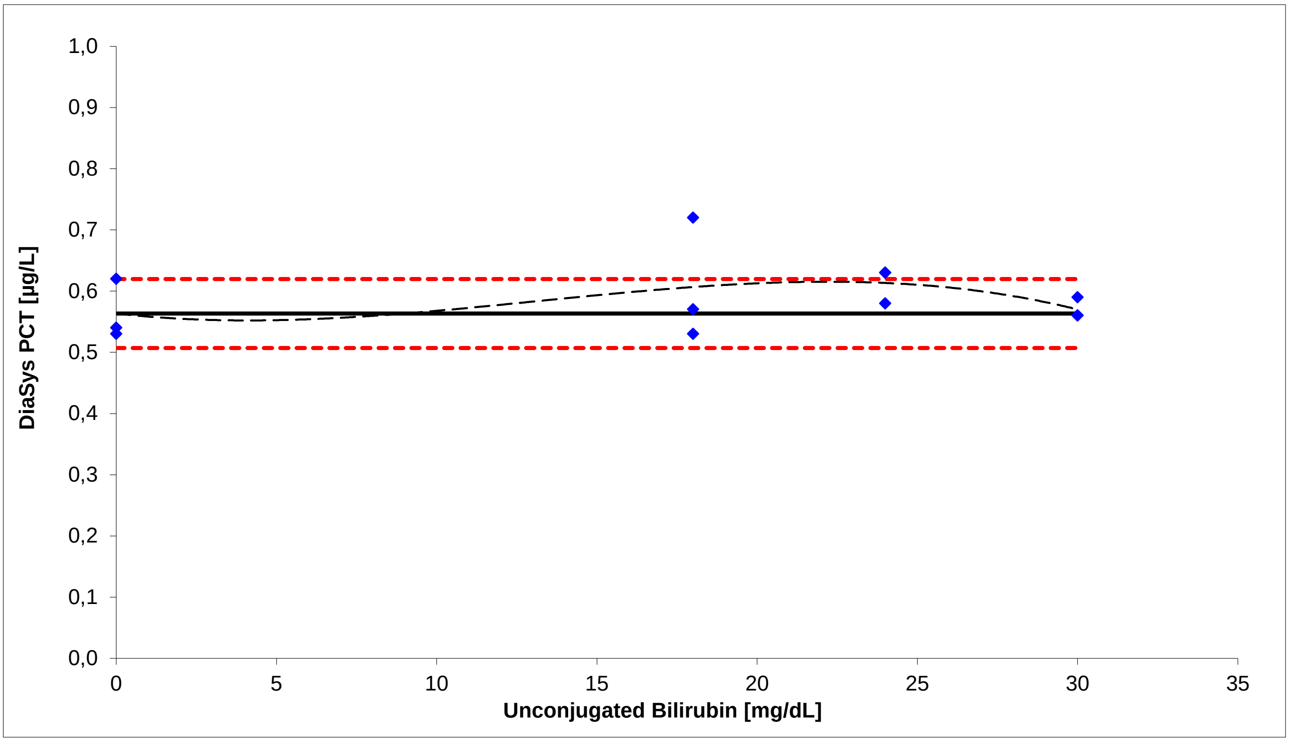

1,0
0,9
0,8
0,7
0,6
0,5
0,4
0,3
0,2
0,1
0,0
DiaSys PCT [µg/L]
0
5
10
15
20
25
30
35
Unconjugated Bilirubin [mg/dL]

F

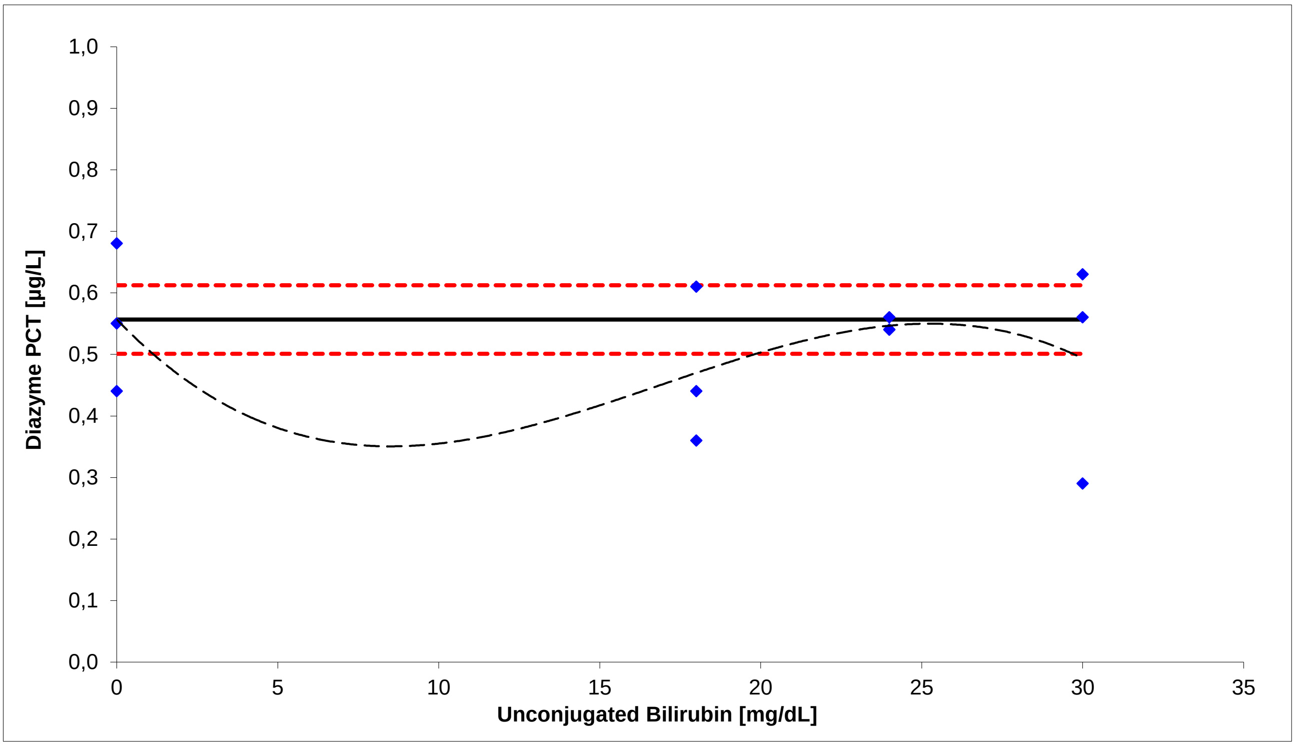

1,0
0,9
0,8
0,7
0,6
0,5
0,4
0,3
0,2
0,1
0,0
Diazyme PCT [µg/L]
0
5
10
15
20
25
30
35
Unconjugated Bilirubin [mg/dL]

G

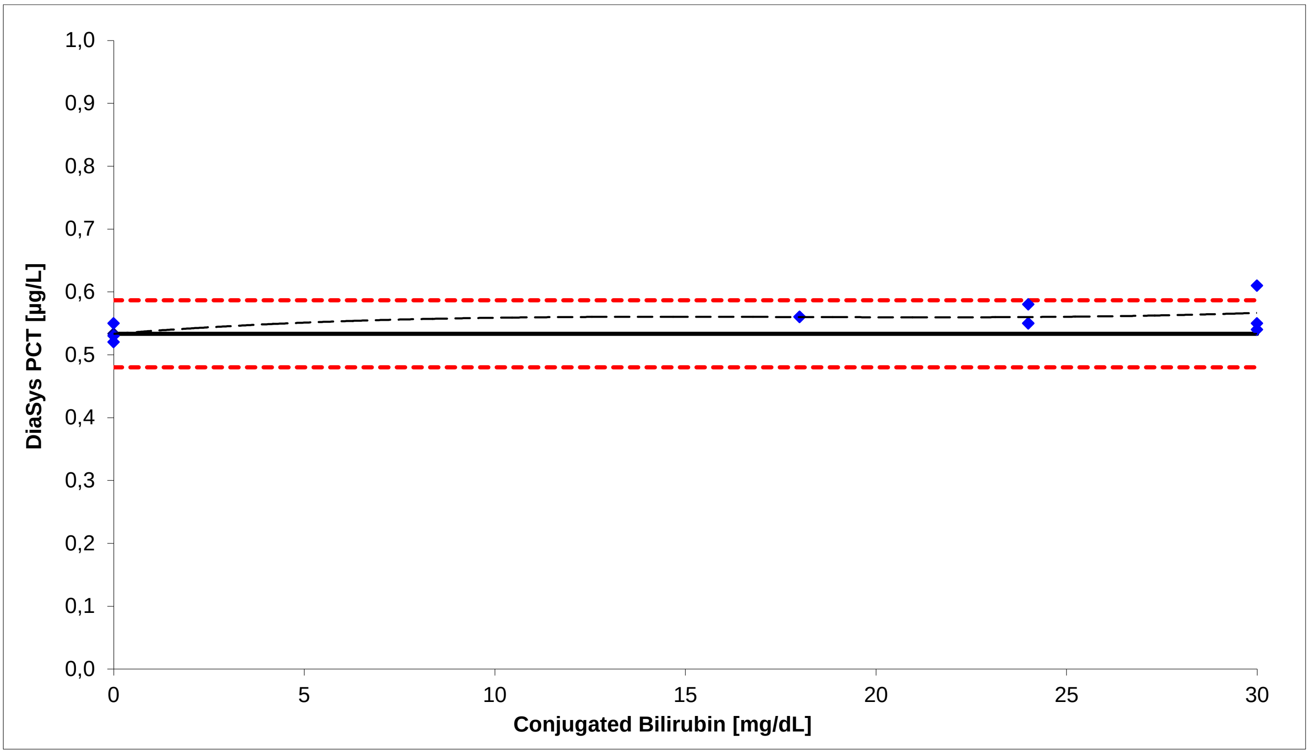
1,0
0,9
0,8
0,7
0,6
0,5
0,4
0,3
0,2
0,1
0,0
0
5
10
15
20
25
30
DiaSys PCT [µg/L]
Conjugated Bilirubin [mg/dL]

H

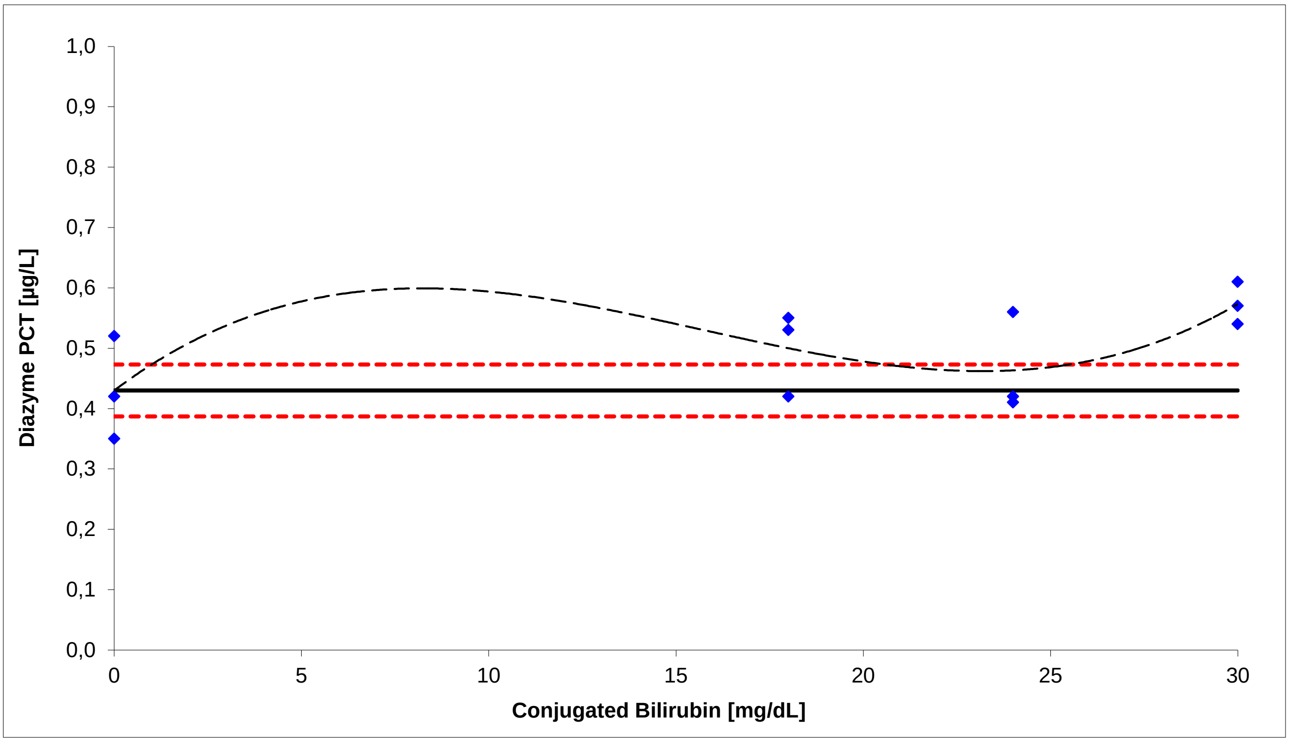
1,0
0,9
0,8
0,7
0,6
0,5
0,4
0,3
0,2
0,1
0,0
0
5
10
15
20
25
30
Diazyme PCT [µg/L]
Conjugated Bilirubin [mg/dL]

I

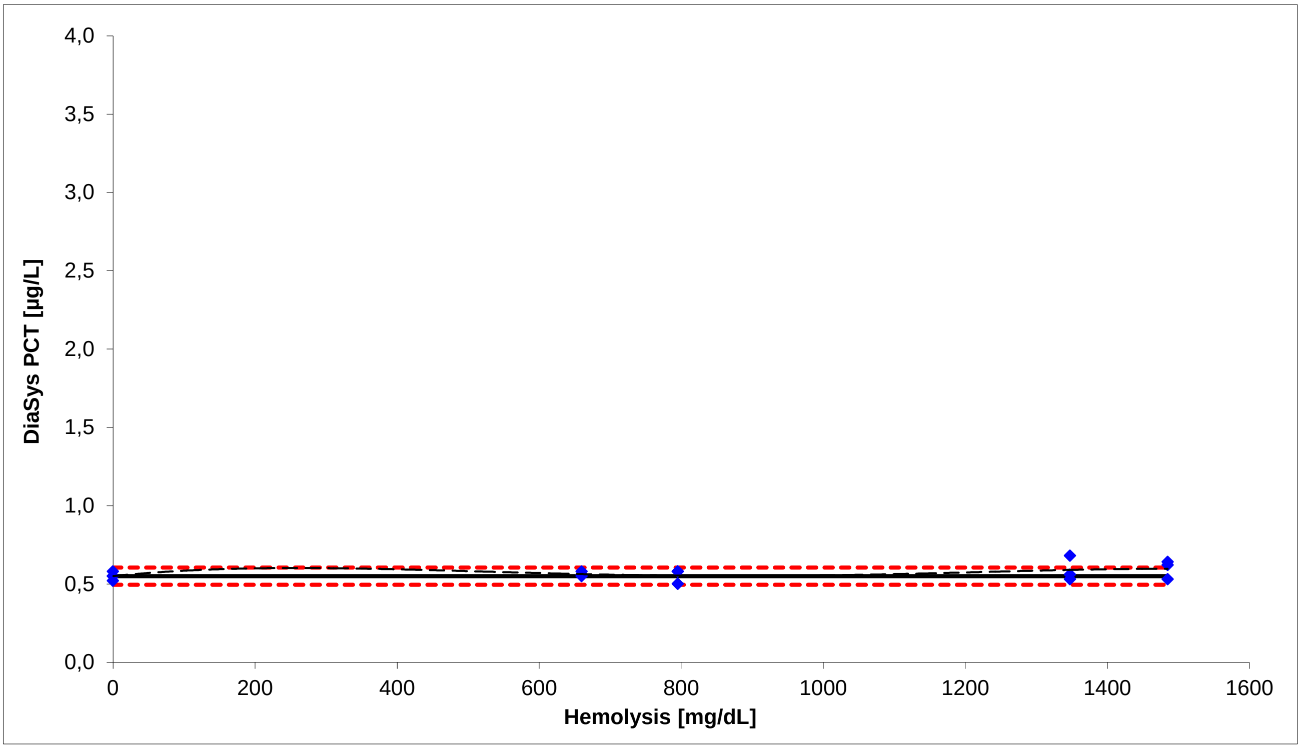

4,0
3,5
3,0
2,5
2,0
1,5
1,0
0,5
0,0
DiaSys PCT [µg/L]
0
200
400
600
800
1000
1200
1400
1600
Hemolysis [mg/dL]

J

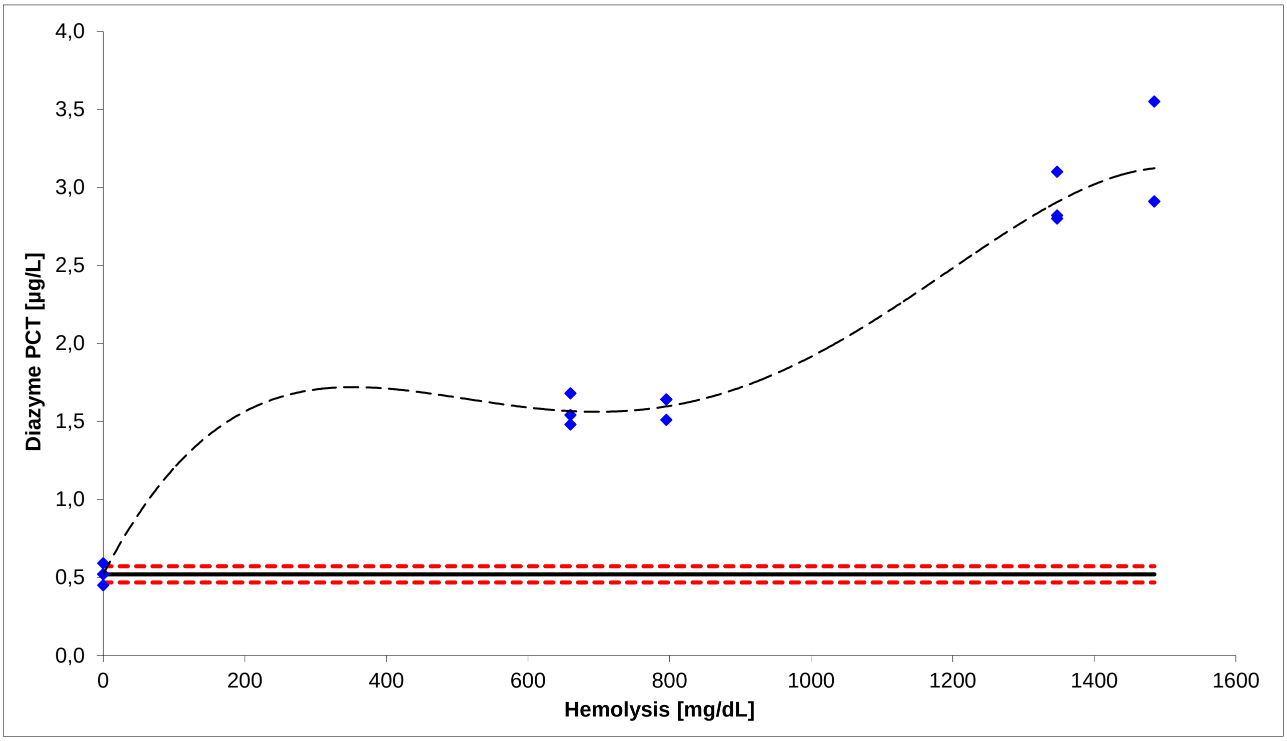

4,0
3,5
3,0
2,5
2,0
1,5
1,0
0,5
0,0
Diazyme PCT [µg/L]
0
200
400
600
800
1000
1200
1400
1600
Hemolysis [mg/dL]
